# Neural Mechanisms Underlying Stop-and-Restart Difficulties: Involvement of the Motor and Perceptual Systems

**DOI:** 10.1371/journal.pone.0082272

**Published:** 2013-11-27

**Authors:** Kentaro Yamanaka, Daichi Nozaki

**Affiliations:** 1 Graduate School of Human Life Sciences, Showa Women's University, Tokyo, Japan; 2 Graduate School of Education, University of Tokyo, Tokyo, Japan; University Medical Center Groningen UMCG, Netherlands

## Abstract

The ability to suddenly stop a planned movement or a movement being performed and restart it after a short interval is an important mechanism that allows appropriate behavior in response to contextual or environmental changes. However, performing such stop-and-restart movements smoothly is difficult at times. We investigated performance (response time) of stop-and-restart movements using a go/stop/re-go task and found consistent stop-and-restart difficulties after short (∼100 ms) stop-to-restart intervals (SRSI), and an increased probability of difficulties after longer (>200 ms) SRSIs, suggesting that two different mechanisms underlie stop-and-restart difficulties. Next, we investigated motor evoked potentials (MEPs) in a moving muscle induced by transcranial magnetic stimulation during a go/stop/re-go task. In re-go trials with a short SRSI (100 ms), the MEP amplitude continued to decrease after the re-go-signal onset, indicating that stop-and-restart difficulties with short SRSIs might be associated with a neural mechanism in the human motor system, namely, stop-related suppression of corticomotor (CM) excitability. Finally, we recorded electroencephalogram (EEG) activity during a go/stop/re-go task and performed a single-trial-based EEG power and phase time-frequency analysis. Alpha-band EEG phase locking to re-go-signal, which was only observed in re-go trials with long SRSI (250 ms), weakened in the delayed re-go response trials. These EEG phase dynamics indicate an association between stop-and-restart difficulties with long SRSIs and a neural mechanism in the human perception system, namely, decreased probability of EEG phase locking to visual stimuli. In contrast, smooth stop-and-restart human movement can be achieved in re-go trials with sufficient SRSI (150–200 ms), because release of stop-related suppression and simultaneous counter-activation of CM excitability may occur as a single task without second re-go-signal perception. These results suggest that skilled motor behavior is subject to various constraints in not only motor, but also perceptual (and attentional), systems.

## Introduction

Ongoing changes in external or internal environments require people to suddenly stop a planned movement or a movement being performed and restart it after a short interval. Such executive function plays a critical role in daily life. However, performing a stop-and-restart movement smoothly is sometimes difficult. This difficulty is typically seen in situations where people momentarily hesitate while trying to decide what to do; for example, when a football or basketball player is immobilized when caught off guard by an opponent's fake motion. These situations highlight the difficulty of stop-and-restart movements under certain conditions.

Human inhibitory control has been generally investigated using a stop-signal paradigm [1]–[4]. Human restart function just after stopping, however, has not been fully investigated. Although psychophysical studies of stop-and-restart performance have been published [5], [6], inconsistent results have been reported: response time (RT) to the restart signal was reported to change nonlinearly [5] or decrease almost linearly [6] with an increase in the stop-to-restart interval (SRSI). One reason for the inconsistent results may be that these studies used different tasks: a timing-coincident go/stop/go paradigm and a go/stop/change reaction time paradigm. Furthermore, none of the studies collected physiological measurements; therefore, the mechanisms underlying stop-and-restart difficulty remain unclear.

A physiological factor underlying stop-and-restart difficulty in the corticomotoneuronal (CM) system might be stop-related suppression of CM excitability. Transcranial magnetic stimulation (TMS) studies have shown that the motor evoked potential (MEP) in a moving muscle, which primarily reflects CM excitability [7], [8], is transiently suppressed during no-go trials in go/no-go tasks [9]–[12], and during stop trials in stop-signal tasks [13], [14]. These studies also reported that CM excitability returned to baseline after the transient suppression for no-go or stop reactions [10]–[13]. However, the effects of stop-related suppression of CM excitability and its return to baseline on immediate restart are still unknown.

Another factor underlying stop-and-restart difficulty might be a decrease in visual perception system function. Several studies have demonstrated that alpha-band (around 10 Hz) electroencephalographic (EEG) power is negatively associated with visual perceptual performance [15]–[18]. In addition, it has been reported that phase locking of alpha-band EEG oscillation around the early event-related potential (ERP) component is associated with visual perception [16], [18]. Therefore, if decreased restart signal perception is related to stop-and-restart difficulty, the difference in restart movement performance might be reflected in EEG power and phase dynamics.

In this study, we first conducted an experiment using a timing-coincident go/stop/re-go task (an extension of the stop-signal paradigm) [5], and reassessed the effect of SRSIs on the mean and standard deviation (SD) of RTs in re-go trials (Experiment 1), which are assumed to reflect stop-and-restart difficulty. Next, we conducted two electrophysiological experiments using TMS (Experiment 2) and surface-EEG recordings (Experiment 3). In the TMS experiment, we examined the effect of stop-related suppression of CM excitability on stop-and-restart difficulty with short SRSIs. In the surface-EEG experiment, we investigated the mechanism underlying stop-and-restart difficulty with long SRSIs using traditional ERP and a single-trial-based EEG power and phase time-frequency (T/F) analysis. These three experiments were conducted with the aim of identifying the mechanisms underlying stop-and-restart difficulties, and we observed involvement of both motor and perceptual systems.

## Methods

### Participants

Fifteen right-handed healthy volunteers (4 women and 11 men, 27.9±5.7 years) participated in Experiment 1; a new group of nine right-handed healthy volunteers (3 women and 6 men, 26.9±4.7 years) participated in Experiment 2; a third group of ten right-handed healthy volunteers (3 women and 7 men, 24.6±5.1 years) participated in Experiment 3. The experimental procedures were approved by the local ethics committee of the Graduate School of Education at the University of Tokyo, and the Graduate School of Human Life Sciences at the Showa Women's University. Written informed consent was obtained from all participants before any experimental procedures were performed.

### Experiment 1

#### Task setting and procedure

Each participant was comfortably seated in a chair facing a 12.1-in. personal computer (PC) display (screen resolution: 1280×800 pixels; refresh rate: 60 Hz). Participants placed their right index finger on the main (left) button of a computer mouse. All participants performed simple reaction time (SRT) tasks and timing-coincident go/stop and go/stop/re-go tasks in a fixed order (SRT, go/stop, go/stop/re-go, go/stop/re-go, go/stop, SRT) because of their hierarchical nature. This order ensured that learning and fatigue were balanced across conditions. Before starting each experimental block, participants performed a practice block consisting of approximately 20 trials of each task. Inter-trial intervals for all tasks were 3 s.

**Figure 1:**
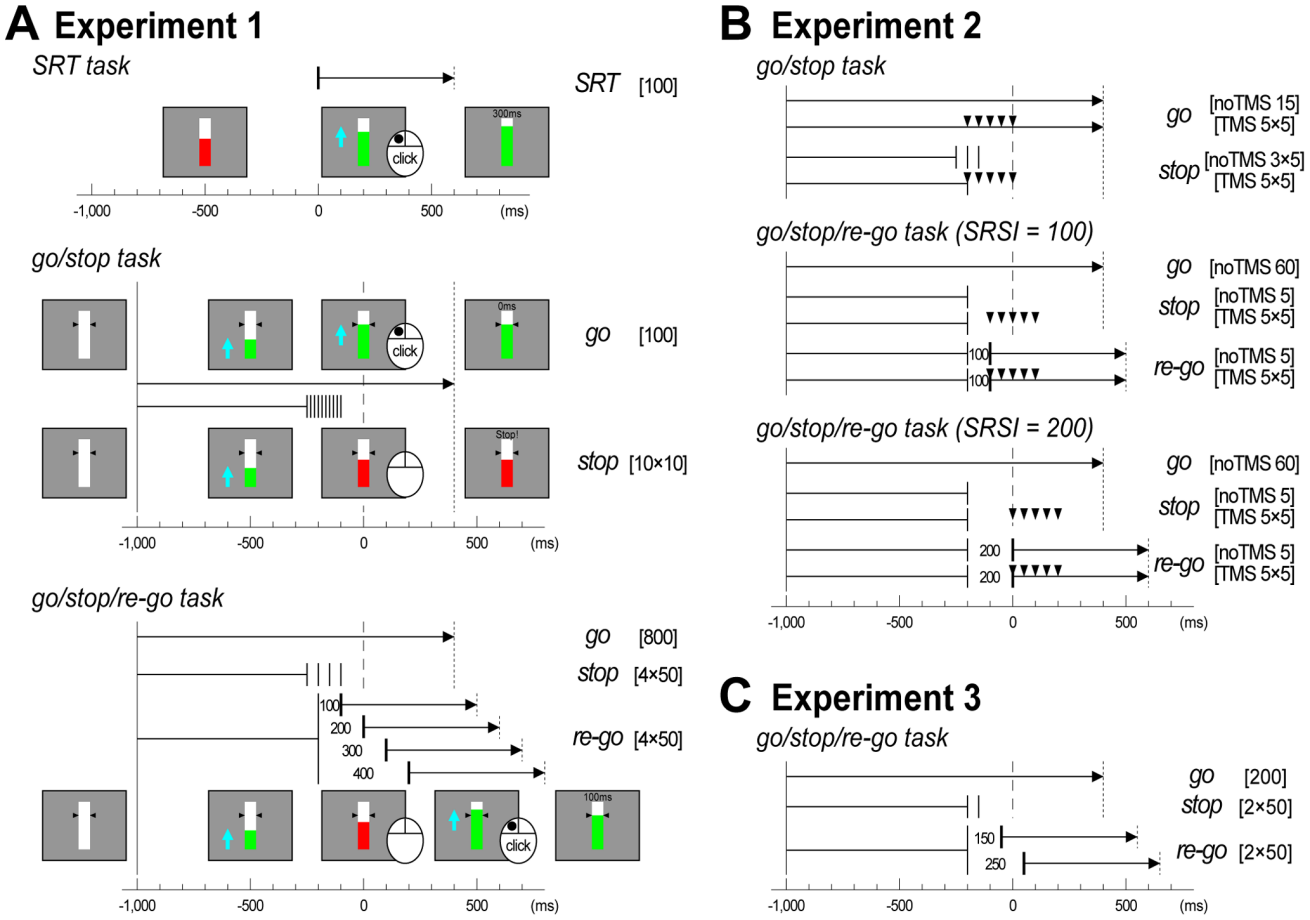
Task designs of the three experiments. (**A**) Illustrations of display and trial structure for the simple reaction time (SRT), go/stop, and go/stop/re-go tasks in Experiment 1. The trial type is noted on the right, and the total number of trials is shown in brackets. Time scale is displayed at the bottom. Vertical lines at −1000 ms represent indicator onset, vertical dashed lines at 0 ms represent the target, and vertical dotted lines represent feedback onset. In stop and re-go trials, the time points at which the indicator stopped are shown with vertical thin bars. In SRT task and re-go trials, time points at which the indicator (re-) started are shown with vertical thick bars. Small numbers between stop and restart bars in re-go trials indicate stop-to-restart intervals (SRSIs) in milliseconds. (**B**) Illustrations of trial structure for the go/stop and go/stop/re-go tasks in Experiment 2. Small triangles represent time points at which transcranial magnetic stimulation was delivered (TMS time). (**C**) Illustration of trial structure for the go/stop/re-go task in Experiment 3.

In the SRT task (Figure 1A, top), each trial began with the presentation of a white bar against a gray background. A red indicator was presented at 8/14 of the height of the white bar at the beginning of the trial. After a variable delay of 900, 1000, 1100, or 1200 ms, the red indicator turned green and moved upward at a constant rate, reaching the top of the bar in 600 ms. Participants were instructed to click the mouse to stop the indicator as fast as possible immediately after the indicator began moving. All participants performed two blocks of 50 trials. There were 100 SRT task trials in total.

In the go/stop task (Figure 1A, middle), each trial began with the presentation of a white bar against a gray background, with two small black triangles indicating the target. After 600 ms, a green indicator moved upward from the bottom of the bar at a constant rate, reaching the target in 1000 ms, and the top of the bar in 1400 ms. Participants were instructed to click the mouse to stop the moving green indicator when it reached the target (referred to as go trials). On half of the trials, the moving green indicator unexpectedly stopped and turned red just before it reached the target. Participants were instructed to withhold their click when the moving green indicator stopped and turned red (referred to as stop trials). For stop trials, the indicator stopped randomly at a time point (stop time: ST) of −250, −233, −217, −200, −183, −167, −150, −133, –117, or −100 ms relative to the target. Participants were informed that the indicator in some trials would be easy to stop, and in others it would be more difficult or impossible to stop because the ST would be too close to the target. All participants performed two blocks of 100 trials. There were 200 trials in total: 100 go trials and 100 stop trials with 10 trials for each ST.

In the go/stop/re-go task (Figure 1A, bottom), re-go trials were included in addition to go and stop trials. In half of the trials in which the green indicator stopped and turned red, the stopped red indicator turned back to green and moved upward again after a short interval (stop-to-restart interval: SRSI). Participants were instructed to click the mouse as fast as possible immediately after the restart (referred to as re-go trials), even though stopping the indicator at the target would be difficult or impossible. For stop trials, the indicator stopped randomly at an ST of −250, −200, −150, or −100 ms to prevent easy prediction of STs. Conversely, to increase the number of successful re-go trials, the indicator stopped consistently at an ST of −200 ms and restarted randomly after an SRSI of 100, 200, 300, and 400 ms on re-go trials. None of the participants noticed this biased ST setting during the experiments. All participants performed two blocks of 400 trials. There were 800 trials in total: 400 go trials, 200 stop trials with 50 trials for each ST, and 200 re-go trials with 50 trials for each SRSI.

After each trial, visual feedback (RT relative to target [ms] or “miss” for go, re-go, and SRT task trials; “stop!!” or “false alarm” with RT [ms] for stop trials) was presented for 500 ms on the central bar. This timing (Figure 1A) was used to prevent eye blinks before the onset of visual feedback.

#### Performance analysis and statistics

RTs in the re-go trials were first transformed into times relative to re-go-signal onset so that they could be compared with RTs in the SRT task trials, because the task demands in the re-go part of the re-go trials are similar to those in the SRT task. Next, outlying RTs were discarded according to the following criteria: <–100 ms and >150 ms in go trials (1.3% for the go/stop task; 2.2% for the go/stop/re-go task) and <150 ms and >400 ms in re-go trials (14.1±1.8% for all 4 SRSIs) and SRT task trials (2.4%). The mean and SD of the RTs were then calculated for each participant and trial condition. For stop trials in the go/stop and go/stop/re-go tasks, percentage of correct responses (%correct) was calculated for each participant and ST. Next, the ST for which the probability of successful stopping was 50% (50%ST) was determined using the least-square fitting curve to the sigmoid function. The 50%ST was subtracted from the mean go RT to determine stop-signal reaction time (SSRT), which is the estimated time required for unobservable stop processes based on a *race model* [1]–[4].

Before assessing task performance in re-go trials, we needed to compare task performance between the go/stop and go/stop/re-go tasks. Therefore, mean go RTs, 50%STs, and SSRTs in the go/stop and go/stop/re-go tasks were compared using paired *t*-tests. In addition, %corrects for the 4 STs (−250, −200, −150, and −100 ms) used in both the go/stop and go/stop/re-go tasks were submitted to a two-way repeated measures analysis of variance (ANOVA) with within-participant factors of task and ST. Next, %corrects in re-go trials with four different SRSIs were submitted to a one-way repeated measures ANOVA with a within-participant factor of SRSIs. In addition, planned comparisons were conducted comparing %correct in stop trials with an ST of –200 ms (control condition) with each of the four SRSI re-go trials (STs were fixed at –200 ms) using Bonferroni-corrected paired *t*-tests.

After these analyses, the mean and SD of re-go RTs with four different SRSIs were submitted to one-way repeated measures ANOVAs with a within-participant factor of SRSI to assess re-go trial performance. The Bonferroni correction for multiple comparisons was used for post hoc *t*-tests. In addition, planned comparisons of means and SDs were conducted comparing SRT task trials (control condition) to each of the four SRSI re-go trials, using Bonferroni-corrected paired *t*-tests. Before all the paired *t*-tests, we confirmed that the differences between pairs of variables are normally distributed. We used a Mauchley's test to evaluate the sphericity assumption of the repeated measures ANOVA, and, if necessary, a Greenhouse-Geisser procedure was used to correct the degrees of freedom. The significance level for all tests was *p*<.05.

### Experiment 2

#### TMS and EMG recording

The procedure in Experiment 2 was identical to Experiment 1, except for the following. Electromyogram (EMG) was recorded from the right first dorsal interosseous (FDI) muscle using Ag/AgCl surface electrodes. The EMG signal was amplified and filtered (bandpass 16–3000 Hz; AB-621B, Nihon Kohden, Tokyo, Japan). TMS was applied using a Magstim 200 and a figure-8-shaped coil (Magstim Co. Ltd., Whitland, UK; maximum output 1.5 T, 7-cm diameters). A mechanical arm and an elastic band were used to keep the coil at the same position and direction against the participant's scalp throughout the experiment. The coil was placed in the optimal position and direction to elicit MEPs in the right FDI muscle. TMS intensity was expressed as a percentage of the motor threshold (MT; 50.6±4.6% of the maximal stimulator output: mean ± SD for all participants), which was defined as the minimum intensity necessary to induce MEPs of >50 µV in the resting right FDI muscle in at least three of five trials. TMS intensity in the experiment was set to suprathreshold (120% of MT) to obtain MEPs, which were stably induced and clearly identified in the resting FDI muscle (1.57±0.25 mV: mean ± SD for all participants). Coil position and MT were repeatedly checked and maintained throughout the experiment. EMG signal and two trigger signals from the PC indicating the onset of the task and TMS were digitized at a sampling rate of 2000 Hz for 1700 ms, starting at −1000 ms relative to the target, and stored for offline analysis.

#### Task setting and procedure

All participants completed one block of the go/stop task followed by two blocks of the go/stop/re-go tasks (in which the SRSI was fixed at 100 and 200 ms, respectively), after practice blocks of approximately 20 trials. Inter-trial intervals for all tasks were 6 s. For the go/stop task (Figure 1B, top), there were 80 trials in total, 40 go and 40 stop, each consisting of 25 trials with TMS (TMS trials) and 15 trials without TMS (no-TMS trials). In both go and stop trials, TMS was randomly delivered at a TMS time of −200, −150, −100, −50, or 0 ms relative to the target, with 5 trials for each TMS time. The indicator stopped randomly at an ST of −250, −200, or −150 ms in the no-TMS stop trials, and stopped consistently at an ST of −200 ms in the TMS stop trials. For the go/stop/re-go tasks with an SRSI of 100 or 200 ms each (Figure. 1B, middle and bottom), there were 120 trials in total: 60 go, 30 stop, and 30 re-go trials. TMS was delivered in only 25 stop trials and 25 re-go trials. In all of the TMS stop and TMS re-go trials, the indicator stopped consistently at an ST of −200 ms, and TMS was randomly delivered at a TMS time of 0, 50, 100, 150, or 200 ms relative to the re-go-signal onset (or corresponding times in the stop trials). There were 5 trials for each TMS time.

#### MEP analysis and statistics

In addition to the mechanical response onset that was detected by clicking the mouse, we defined EMG onset as the first time point at which the EMG signal crossed threshold levels (defined individually for each participant; 3-5 SDs during 50 ms before the TMS onset at rest) in the time window from 300 ms before the target to feedback onset. Then, go and re-go trials with outlying RTs and stop and re-go trials with false alarm responses were determined by applying the same criteria as Experiment 1 to the EMG onset. Finally, TMS trials with an EMG onset before the TMS time were excluded from the MEP analysis. However, these exclusion criteria did not apply to go and re-go trials with TMS at the last two TMS time points, because the EMG bursts had already started at these time points in most of the correct (successful) go or re-go trials. After data rejection, peak-to-peak MEP amplitudes and root-mean-square EMGs (rmsEMGs) during 50 ms before the TMS onset were measured for each trial with TMS. Mean MEP amplitudes and mean rmsEMGs were calculated for each participant, task, and TMS time (at least 3 trials per condition). Finally, group mean MEP amplitudes and group mean rmsEMGs were calculated for each task and TMS time.

To evaluate TMS time-dependent modulation of mean MEP amplitudes, Friedman tests were applied for each trial condition. In addition, planned comparisons were conducted comparing the mean MEP amplitudes at each TMS time and mean MEP amplitudes at rest using Bonferroni-corrected Wilcoxon signed-rank tests. We also assessed mean rmsEMGs in the same way as mean MEP amplitudes. Next, to assess MEP amplitude modulation during re-go trials with different SRSIs with reference to the re-go-signal onset, mean MEP amplitudes in re-go trials were rearranged according to TMS time relative to re-go-signal onset. Finally, planned comparisons for each TMS time were conducted using Bonferroni-corrected Wilcoxon signed-rank tests. The significance level for all tests was *p*<.05.

### Experiment 3

#### EEG recording

The procedure in Experiment 3 was identical to Experiment 1, except for the following. EEG was recorded from 21 electrodes. Nineteen tin electrodes were mounted on an elastic cap (Electro-Cap International Inc., Eaton, OH) corresponding to the International 10–20 System of electrode placement, and two additional electrodes were attached to the left and right ears. Data were recorded against a reference placed at AFz and later re-referenced offline to averaged earlobes. An electrooculogram (EOG) was recorded with a pair of electrodes placed above and beside the left eye. Electrode impedance was maintained below 10 kΩ. Using an EEG recording system (Neurofax EEG-1100; Nihon Kohden, Tokyo, Japan), EEG and EOG signals were amplified, filtered (bandpass settings: 0.5–100 Hz), and continuously stored with a trigger signal from the PC indicating task onset at a sampling rate of 500 Hz for offline analysis.

#### Task setting and procedure

Participants performed eight blocks of the go/stop/re-go task, each consisting of 50 trials, preceded by practice blocks of approximately 20 trials for each task. For the go/stop/re-go tasks (Figure 1C), there were 400 trials in total: 200 go, 100 stop, and 100 re-go. In stop trials, the indicator stopped randomly at an ST of −200 or −150 ms, with 50 trials for each ST. In re-go trials, it stopped consistently at an ST of −200 ms, and restarted randomly after an SRSI of 150 or 250 ms, with 50 trials for each SRSI [re-go(150) and re-go(250)].

#### EEG analysis and statistics

Continuous EEG data of the 19 channels were segmented offline into epochs within ±1000 ms of the target. Go and re-go trials with outlying RTs and stop and re-go trials with false alarm responses (determined using the criteria outlined in Experiment 1) were excluded from EEG analysis (0.1% of go trials, 14.6% of stop trials and 12.6% of re-go trials for all participants). Trials including large potentials (>±100 µV) generated by eye blink, eye movement, muscle activity, or external noise, were also excluded from EEG analysis (4.2% of go trials, 2.3% of stop trials, and 3.6% of re-go trials for all participants).

For traditional ERP analysis, we first averaged the EEG data from the 19 channels separately for each participant and task condition. We then obtained 19 grand mean ERPs. Next, for the T/F analysis of EEG power and phase dynamics [19]–[23], we convolved the EEG data from the 19 channels with complex 4-cycle Morlet wavelets. Their central frequencies were changed from 4 to 7 Hz (theta band) in 0.5-Hz steps, from 8 to 14 Hz (alpha band) in 1-Hz steps, and from 16 to 28 Hz (beta band) in 2-Hz steps. The instantaneous power ***p*** *_k_* (***t*** *,* ***f***) = RE(***w*** *_k_* [***t*** *,* ***f***])^2^+IM(***w*** *_k_* [***t*** *,* ***f***])^2^ and instantaneous phase ***θ*** *_k_* (***t*** *,* ***f***) =  arctan { ***IM***(***w*** *_k_* [***t*** *,* ***f***])/***RE***(***w*** *_k_* [***t*** *,* ***f***]) } (where ***RE*** and ***IM*** symbolize the real and imaginary parts of a complex number, respectively) were extracted from the wavelet transformed signal ***w*** *_k_* (***t*** *,* ***f***) of trial ***k*** at time ***t*** and frequency ***f***. Using the instantaneous power ***p*** *_k_* (***t*** *,* ***f***) and phase ***θ*** *_k_* (***t*** *,* ***f***), we obtained the event-related power (ERPow) and phase locking index (PLI) of the 19 channels for each participant and task condition, as follows:
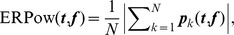




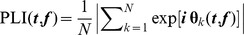
 (*N* =  trial number; 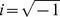).

A PLI close to 1 reflects high phase consistency across trials, whereas a PLI close to 0 reflects high phase variability across trials.

We first compared the EEG data in stop trials with those in re-go(150) and re-go(250) trials (corresponding to short and long SRSI, respectively). Second, we divided the EEG data in the re-go(250) trials into two groups on the basis of the median RT for each participant and compared them [fast re-go(250) and slow re-go(250)]. In these analyses, we obtained the ERPow and PLI within ±500 ms of the target and drew T/F images of ERPow and PLI for each subject and task condition. To assess inter-condition differences in T/F images of the ERPow and PLI, we first conducted a paired *t*-test for each T/F point (*p*<.05, uncorrected). Next, to avoid false positives from multiple comparisons, we applied a procedure for controlling false discovery rate (FDR, q*<.05) [24] to the T/F *p*-value images of each channel. In all statistical results (both corrected and uncorrected), the effects that were only significant in a few neighboring points (less than 100 T/F points after the FDR control) were excluded from interpretation.

## Results

### Experiment 1

**Figure 2:**
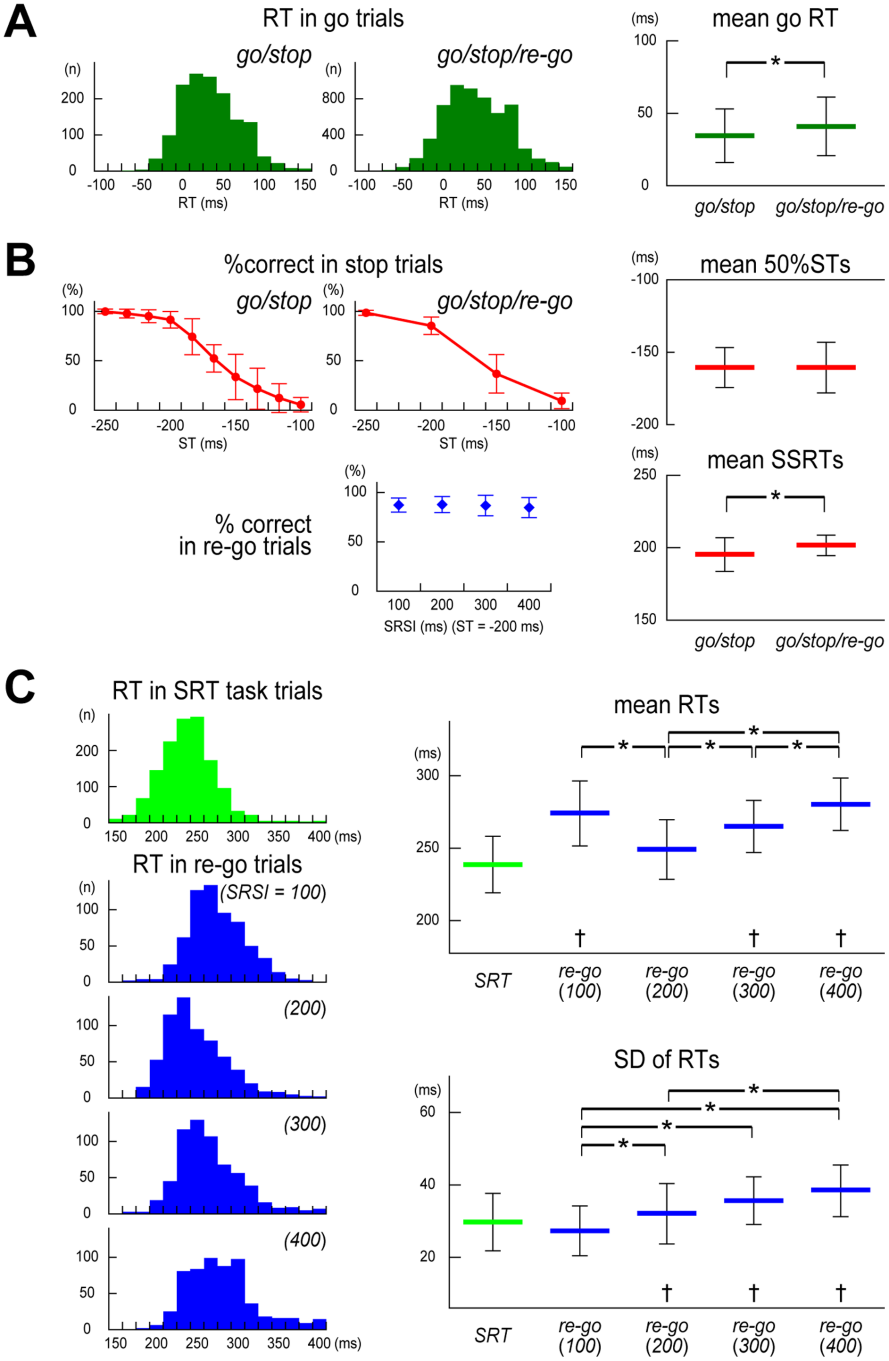
Results of performance analysis in Experiment 1. (**A**) Distributions of response time (RT) in go trials obtained from all 15 subjects (left: go/stop task; middle: go/stop/re-go task) and group mean RTs in go trials (right). RT denotes clicking time relative to the target. (**B**) Group results for the percentage of the correct responses (%correct) in stop trials for each stop time (ST; top left, go/stop task; top center, go/stop/re-go task), group results for %correct in re-go trials for each SRSI (bottom center), and group results of estimated 50%STs and stop-signal reaction times (SSRTs) in go/stop and go/stop/re-go tasks (right). The 50%ST is an estimated stop time in which the probability of successful stopping was 50%. SSRT is the estimated time required for an unobservable stop process. (**C**) RT distributions for the SRT task and re-go trials with four SRSIs obtained from all 15 subjects (left) and group mean and standard deviation (SD) of RTs in the SRT task and re-go trials with four different stop-to-restart intervals (SRSIs, right). RT denotes clicking time from SRT-signal or re-go-signal onset. Error bars show SD. * *p*<.05; significant difference in post hoc analyses. † *p*<.05; significant difference in planned comparisons between RTs in re-go trials for each SRSI and control (SRT trials).

Mean go RTs were 34.3±18.0 ms in the go/stop task and 40.9±20.0 ms in the go/stop/re-go task. And they were more successful at stopping in stop trials the longer the time to the target (Figure 2B, upper left and middle). These results are typical of go and stop trials in the go/stop (or stop-signal) task [1]–[4], [25], .

A two-way repeated measures ANOVA on %corrects in stop trials revealed significant main effects of ST (*F* [3], [42] = 360.7, *p*<.001), but there was no significant main effect of task, and no significant interaction between ST and task. A paired *t*-test revealed no significant difference between the go/stop (–160.8±13.7 ms) and the go/stop/re-go (–160.9±17.5 ms) tasks for 50%STs (Figure 2B, top right). These results indicate that stop trial performance did not differ between the go/stop and the go/stop/re-go tasks. Conversely, mean go RTs in the go/stop task were significantly closer to the target than those in the go/stop/re-go tasks (*t* [14] = 2.84, *p* = .006; Figure 2A, right), and therefore, SSRTs in the go/stop task (195.1±11.6 ms) were significantly smaller than those in the go/stop/re-go tasks (201.8±7.3 ms) (*t* [14] = 2.42, *p* = .015; Figure 2B, bottom right). These results indicate that go trial performance differed between the go/stop and the go/stop/re-go tasks, which might be related to resource competition between the primary go process and the stop process with sub-options (hold or re-go) [2]. Next, a one-way repeated measures ANOVA on %corrects in re-go trials revealed no significant main effect of SRSI (Figure 2B, bottom middle). In addition, planned comparisons revealed that %corrects in re-go trials for each of the four SRSIs were not significantly different from those in stop trials with ST of –200 ms (Figure 2B, middle). These results provide strong support for our hypothesis that, in most re-go trials, participants performed the re-go response just after stopping a planned motor response.

A large proportion of RTs relative to re-go-signal onset in re-go trials were larger than 200 ms (Figure 2C, left). The results indicate that, in most re-go trials, participants could not click at the target, and had to click behind the target. However, the shapes of the re-go RT distributions appear to change considerably across the four SRSIs (Figure 2C, left).

A one-way repeated measures ANOVA on re-go mean RTs revealed a significant main effects of SRSIs (*F* [3], [42] = 24.7, *p*<.001; Figure 2C, top right). Post-hoc analyses showed that mean RTs in re-go trials with an SRSI of 200 ms (248.6±21.0 ms) were significantly shorter than those with an SRSI of 100 ms (274.0±22.6 ms) (*t* [14] = −9.17, *p*<.001), 300 ms (264.9±18.5 ms) (*t* [14] = −4.36, *p*<.001), and 400 ms (280.2±18.5 ms) (*t* [14] = −9.40, *p*<.001), and that mean RTs in re-go trials with an SRSI of 300 ms were significantly shorter than those with an SRSI of 400 ms (*t* [14] = −4.56, *p*<.001). A one-way repeated measures ANOVA on SDs of re-go RTs also revealed a significant main effects of SRSIs (*F* [3], [42] = 8.35, *p*<.001; Figure 2C, bottom right). Post-hoc analyses showed that SDs of re-go RTs with an SRSI of 100 ms (27.3±6.9 ms) were significantly smaller than those with an SRSI of 200 ms (32.0±8.4 ms) (*t* [14] = −3.39, *p* = .004), 300 ms (35.6±6.7 ms) (*t* [14] = −3.64, *p* = .002), and 400 ms (38.4±7.1 ms) (*t* [14] = −7.01, *p*<.001), and that SDs of re-go RTs with an SRSI of 200 ms were significantly smaller than those with an SRSI of 400 ms (*t* [14] = −3.51, *p* = .003).

Planned comparisons (Bonferroni corrected paired *t*-tests) between mean RTs in the re-go trials for each of the four SRSIs and those of the SRT task trials showed that mean SRT task RTs (238.6±19.5 ms) were significantly faster than those of re-go trials with the shortest (100 ms) and two longest (300 and 400 ms) SRSIs (all *ps*<.05), but not significantly different from re-go trials with the second shortest (200 ms) SRSI (Figure 2C, top right). Conversely, the SDs of RTs in the SRT task trials (29.7±7.9 ms) were significantly smaller than those of re-go trials with the three longest (200, 300, and 400 ms) SRSIs (all *ps*<0.05), but were not significantly different from re-go trials with the shortest (100 ms) SRSI (Fig. 2C, bottom right).

### Experiment 2

Since RTs measured in the TMS trials changed due to the effects of TMS (an appearance of MEP and a silent period), we only used no TMS trials to assess task performance. Mean go RTs were 38.7±9.9 ms in the go/stop task, 39.9±12.6 ms in the go/stop/re-go task (SRSI 100 ms), and 43.2±13.0 ms in the go/stop/re-go task (SRSI 200 ms). In the go/stop task, the longer the time to the target, the higher the %correct on stop trials (ST 250 ms: 98.1±5.6%; 200 ms: 79.8±9.9%; 150 ms: 24.1±23.7%). In the go/stop/re-go tasks, %correct of re-go trials (SRSI 100 ms: 84.8±15.7%; SRSI 200 ms: 80.8±8.4%) were almost the same as those of stop trials (SRSI 100 ms: 86.8±7.6%; SRSI 200 ms: 78.8± 15.7%). In contrast, re-go RTs (from re-go-signal onset) changed significantly depending on SRSI (SRSI 100 ms: 288.8±14.3 ms; 200 ms: 265.4±7.1 ms; *t* [9] = 2.81, *p* = .022). Although the number of no-TMS trials was small (15 for go and stop trials in the go/stop task, 5 for stop and re-go trials in the go/stop/re-go tasks, for each participant), these results indicate that task performance in Experiment 2 was similar to that in Experiment 1.

**Figure 3:**
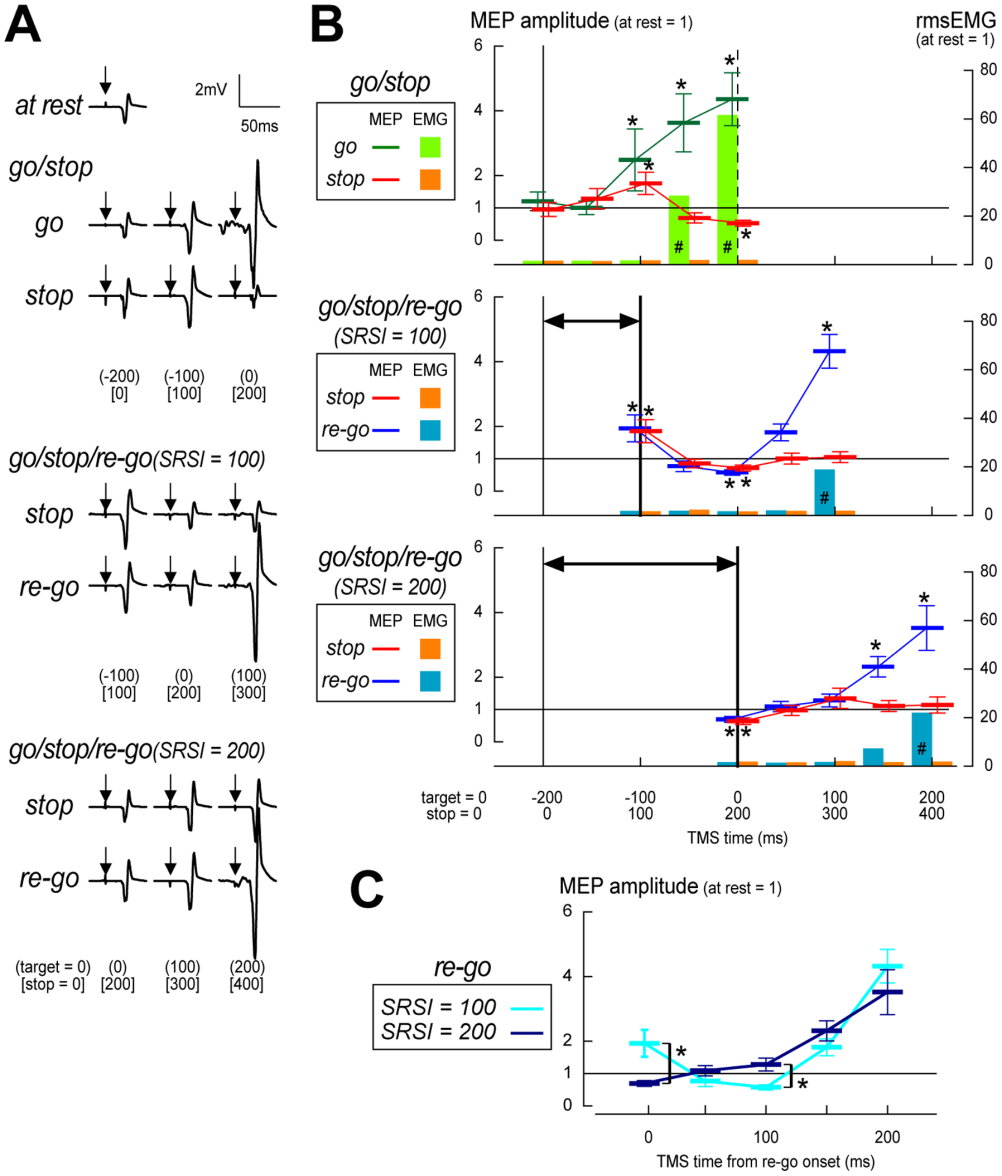
Results of motor evoked potential analyses in Experiment 2. (**A**) Raw motor evoked potential (MEP) waveforms for a typical subject (top: at rest; second from the top: go/stop task; third from the top: go/stop/re-go task with a stop-to-restart interval (SRSI) of 100 ms; bottom: go/stop/re-go task with an SRSI of 200 ms). Trial type is labeled on the right side of the EMG trace. Time point at which transcranial magnetic stimulation was delivered (TMS time) is shown in round bracket (relative to the target) and in square bracket (after stop-signal onset) on the bottom. Arrows on the electromyogram (EMG) traces indicate artifacts from the TMS pulses. (**B**) Group means (±SD) of standardized MEP amplitudes and root-mean-square EMGs (rmsEMGs) (top: go/stop task; middle: go/stop/re-go task with an SRSI of 100 ms; bottom: go/stop/re-go task with an SRSI of 200 ms). Two time scales are displayed at the bottom. Vertical dashed lines at 0 ms in the top panel represent the time point of the target for the primary go task. Vertical thin lines at –200 ms in all three panels represent the time point at which the indicator stopped. Vertical thick lines at –100 ms in the middle panel and at 0 ms in the bottom panel represent time points when the indicator restarted. Two-headed arrows in the middle and bottom panels represent SRSIs in milliseconds. TMS times refer to the time that TMS was delivered relative to the target (top) and after stop-signal onset (bottom). * *p*<.05; significant difference in planned comparisons between MEP amplitudes at each TMS time and baseline (at rest). # *p*<.05; significant difference in planned comparisons between rmsEMGs at each TMS time and baseline (at rest). (**C**) Group mean (±SD) of MEP amplitudes in re-go trials, rearranged according to TMS time relative to re-go-signal onset. * *p*<.05; significant difference in planned comparisons between MEP amplitudes in re-go trials with an SRSI of 100 ms and 200 ms for each TMS time.

In go/stop task TMS trials, as the TMS time approached the target, the MEP amplitude in go trials gradually increased, whereas those in stop trials slightly increased and then decreased (typical raw data: Figure 3A, group result: Figure 3B). Friedman tests on mean MEP amplitudes for go and stop trials revealed significant effects of TMS times (go: *χ* ^2^ [4] = 33.0, *p*<.001; stop: *χ* ^2^ [4] = 17.5, *p* = .002). Planned comparisons (Bonferroni-corrected Wilcoxon signed-rank tests) revealed that, during go trials, the mean MEP amplitudes at TMS times of −100, −50 and 0 ms relative to the target (100, 150, and 200 ms after stop-signal onset) were significantly larger than mean MEP amplitudes at rest (all *ps*<.05). During stop trials, mean MEP amplitudes at a TMS time of −100 ms relative to the target (100 ms after stop-signal onset) were significantly larger, and mean MEP amplitudes at a TMS time of 0 ms relative to the target (200 ms after stop-signal onset) were significantly smaller, than mean MEP amplitudes at rest (*ps*<.05). On the other hand, Friedman tests on mean rmsEMGs revealed significant effects of TMS times for go trials but not for stop trials. Planned comparisons revealed that, during go trials, mean rmsEMGs at TMS times of –50 and 0 ms relative to the target (150, and 200 ms after stop-signal onset) were significantly larger than mean rmsEMGs at rest (all *ps*<.05). These results are in agreement with previous studies using stop-signal tasks [13], [14].

In go/stop/re-go task TMS trials with SRSIs of 100 and 200 ms, the MEP amplitudes in both stop and re-go trials initially increased but later decreased, after which MEP amplitudes in re-go trials abruptly increased, whereas those in stop trials slightly increased (typical raw data: Figure 3A; group result: Figure 3B). Friedman tests on mean MEP amplitudes for both stop and re-go trials with an SRSI of 100 ms revealed significant effects of TMS times (stop: *χ* ^2^ [4] = 16.3, *p* = .003; re-go: *χ* ^2^ [4] = 30.5, *p*<.001). Planned comparisons (Bonferroni-corrected Wilcoxon signed-rank tests) showed that, during stop trials, mean MEP amplitudes at TMS times of −100 and 100 ms relative to the target (100 and 300 ms after stop-signal onset) were significantly larger, and mean MEP amplitudes at a TMS time of 0 ms relative to the target (200 ms after stop-signal onset) were significantly smaller, than mean MEP amplitudes at rest (all *ps*<.05). During re-go trials, mean MEP amplitudes at TMS times of −100 ms relative to the target (100 ms after stop-signal onset) were significantly larger, and mean MEP amplitudes at a TMS time of 0 ms relative to the target (200 ms after stop-signal onset) were significantly smaller, than mean MEP amplitudes at rest (*ps*<.05). On the other hand, Friedman tests on mean rmsEMGs revealed significant effects of TMS times for re-go trials with an SRSI of 100 ms but not for stop trials with an SRSI of 100 ms. Planned comparisons revealed that, during re-go trials, mean rmsEMGs at TMS times of 100 ms relative to the target (200 ms after stop-signal onset) were significantly larger than mean rmsEMGs at rest (*p*<.05). Next, Friedman tests on mean MEP amplitudes for both stop and re-go trials with an SRSI of 200 ms also revealed significant effects of TMS times (stop: *χ* ^2^ [4] = 23.7, *p* = .002; re-go: *χ* ^2^ [4] = 33.0, *p*<.001). Planned comparisons (Bonferroni-corrected Wilcoxon signed-rank tests) showed that, during stop trials, mean MEP amplitudes at a TMS time of 0 ms relative to the target (200 ms after stop-signal onset) were significantly smaller, and mean MEP amplitudes at TMS times of 150 and 200 ms relative to the target (350 and 400 ms after stop-signal onset) were significantly larger, than mean MEP amplitudes at rest (all *ps*<0.05). During re-go trials, the mean MEP amplitudes at TMS times of 0 ms relative to the target (200 ms after stop-signal onset) were significantly smaller than mean MEP amplitudes at rest (*p*<.05). On the other hand, Friedman tests on mean rmsEMGs revealed significant effects of TMS times for re-go trials with an SRSI of 200 ms but not for stop trials with an SRSI of 200 ms. Planned comparisons revealed that, during re-go trials, mean rmsEMGs at TMS times of 200 ms relative to the target (200 ms after stop-signal onset) were significantly larger than mean rmsEMGs at rest (*p*<.05).

After rearrangement of TMS times relative to re-go-signal onset, gradual MEP increases were observed in re-go trials with SRSIs of both 100 and 200 ms (Figure 3C). For each TMS time relative to re-go-signal onset, planned comparison (Bonferroni-corrected Wilcoxon signed-rank tests) revealed that, at a TMS time of 0 ms after re-go-signal onset, mean MEP amplitudes in re-go trials with SRSIs of 100 ms were significantly larger than those in re-go trials with SRSIs of 200 ms (*p*<.05). In contrast, at a TMS time of 100 ms after re-go-signal onset, mean MEP amplitudes in re-go trials with SRSIs of 100 ms were significantly smaller than those in re-go trials with SRSIs of 200 ms (*p*<.05).

### Experiment 3

Mean go RTs were 40.6±10.0 ms. The longer the time to the target, the higher %correct of stop trials (ST 200 ms: 85.4±8.8%; 150 ms: 38.9±14.1%). Moreover, accuracy was almost equal between re-go trials with the two different SRSIs (SRSI 150 ms: 87.1±7.3%; 250 ms: 87.4±7.8%), but re-go RTs significantly changed depending on SRSI (150 ms: 269.8±22.8 ms; 250 ms: 291.8±23.9 ms; *t* [9] = −5.89, *p*<.001). These results indicate that task performance in Experiment 3 was similar to those in Experiments 1 and 2.

**Figure 4:**
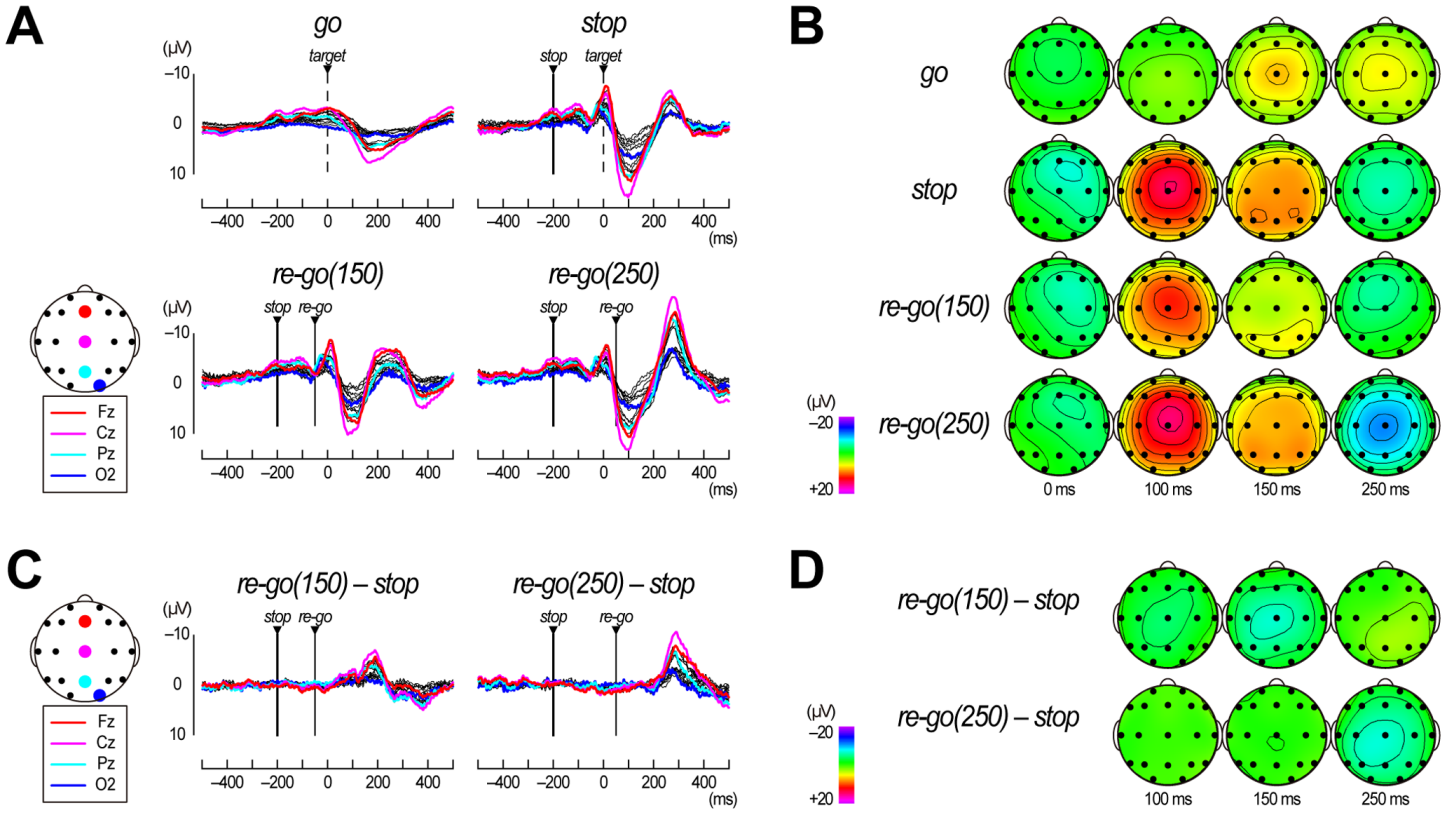
Results of event-related potential analysis in Experiment 3. (**A**) Grand mean event-related potential (ERP) waveforms obtained during go trials, stop trials, re-go trials with an SRSI of 150 ms (re-go(150)), and re-go trials with an SRSI of 250 ms (re-go(250)). The waveforms at Fz, Cz, Pz, and O2 are shown as thick, colored lines, and the waveforms at the other sites are shown as thin, black lines. Vertical thin lines represent stop-signal onset, vertical dashed lines represent target time for the primary go trials, and vertical thick lines represent re-go-signal onset. Time scales relative to the target are displayed at the bottom. (**B**) Scalp topographies of grand mean ERPs in stop, re-go(150), and re-go(250) trials. They are displayed only at 0, 100, 150, and 250 ms relative to the target. The ERP difference topography of re-go(150) – stop are only displayed for 150 ms, and that of re-go(250) – stop are only displayed for 250 ms. Both time points correspond to 200 ms after re-go-signal onset. (**C**) Grand mean ERP difference waveforms (re-go(150) – stop and re-go(250) – stop). Vertical lines and time scale are displayed in the same manner as (A). (**D**) Scalp topographies of grand mean ERP differences (re-go(150) – stop and re-go(250) – stop). They are displayed at 100, 150, and 250 ms relative to the target.

When we compared grand mean ERP waveforms in go, stop, re-go(150), and re-go(250) trials (Figure 4A), negative-positive ERP deflections after stop-signal onset were observed in stop and two re-go conditions. The negative-positive ERP deflections over the frontocentral areas corresponded to N200 and P300 after stop-signal onset (Figure 4B, 0 and 100 ms), which is consistent with the results of previous studies using stop-signal tasks [3], [27]. After re-go-signal onset, the waveforms in both re-go(150) and re-go(250) trials separated from those in stop trials (Figure 4B, 150 and 250 ms). Therefore, we observed negative deflections in the grand mean ERP difference waveforms from about 100 ms in re-go(150) – stop waveforms and from about 200 ms in re-go(250) – stop waveforms, both of which correspond to about 150 ms after re-go-signal onset (Figure 4C, D). These results indicate that the re-go–specific ERP components appeared after the onset of the re-go-signal.

**Figure 5:**
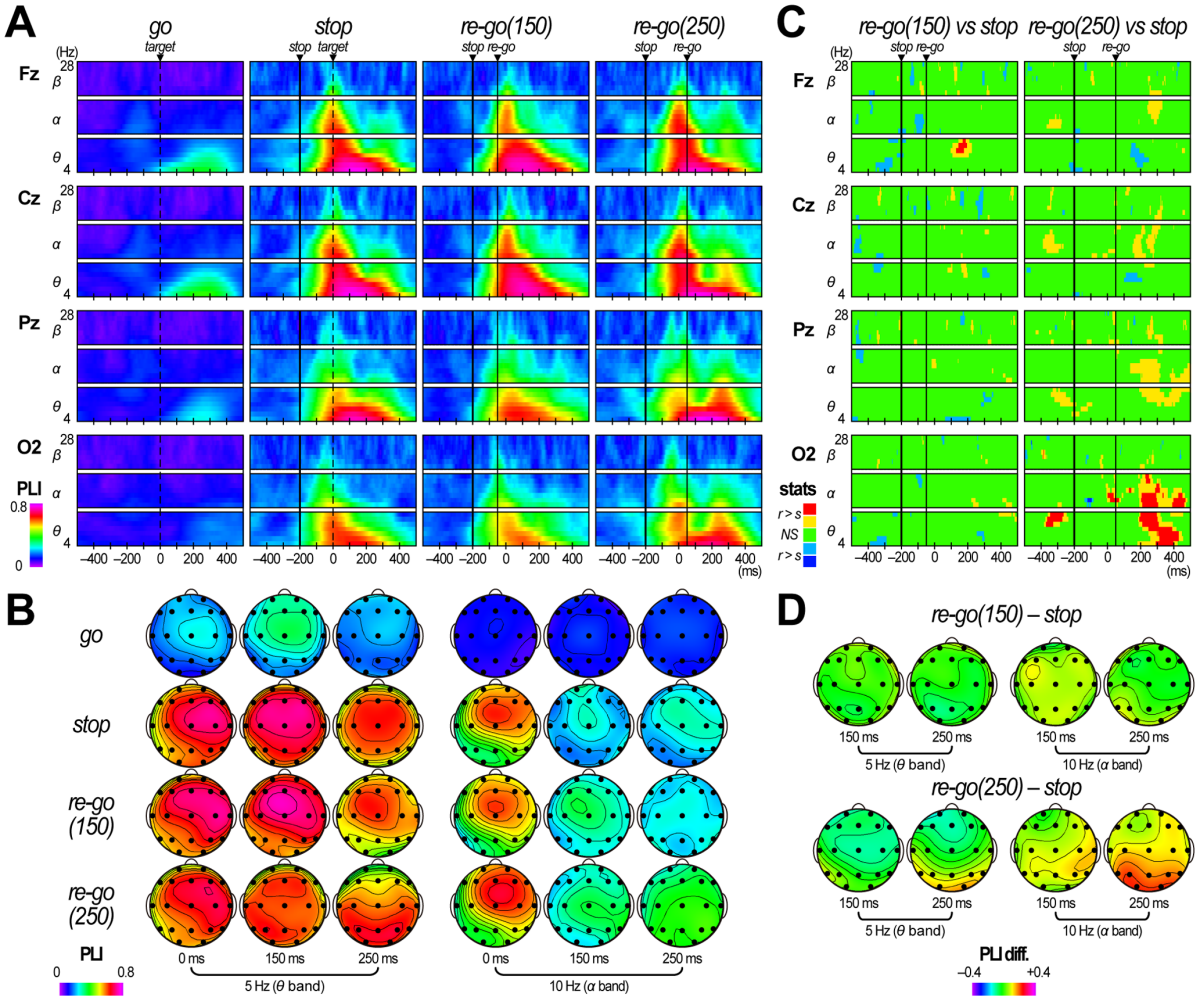
Results of phase locking index analysis in Experiment 3. (**A**) Time/frequency (T/F) images of grand mean phase locking index (PLI) obtained during go trials, stop trials, re-go trials with an SRSI of 150 ms (re-go(150)), and re-go trials with an SRSI of 250 ms (re-go(250)). The T/F images are shown only at Fz, Cz, Pz, and O2. Vertical thin lines represent stop-signal onset, vertical dashed lines represent target time for the primary go trials, and vertical thick lines represent re-go-signal onset. Time scale is displayed at the bottom. (**B**) Scalp topographies of grand mean PLI in go, stop, re-go(150), and re-go(250) trials. They are displayed at time/frequency points described in the figure. (**C**) T/F images indicating the results of the PLI statistical test (re-go(150) vs. stop and re-go(250) vs. stop). The T/F images are only shown at Fz, Cz, Pz, and O2. The yellow and blue parts of the T/F images represent significant differences (*p*<.05; paired *t*-test), and the red and blue parts of the T/F images represent significant differences after correction for multiple comparisons (*p*<.05; paired *t*-test with FDR control; q*<.05). (**D**) Scalp topographies of grand mean PLI differences (re-go(150) – stop and re-go(250) – stop). They are displayed only at some time/frequency points described in the figure.

When we compared grand mean PLI T/F images in go, stop, re-go(150), and re-go(250) trials, identical transient theta-to-alpha-band PLI increases were observed over the frontocentral areas around 0 ms (corresponding to 200 ms after stop-signal onset), in stop and both re-go conditions (Figure 5A, B), indicating the existence of stop-related EEG phase locking across trials. In addition, we observed a second transient theta-to-alpha-band PLI increase over the parieto-occipital sites during about 200–300 ms (corresponding to 150–250 ms after re-go-signal onset) only in re-go(250) trials (Figure 5B, D). Consequently, the theta-to-alpha-band PLI values at O2 in re-go(250) trials were significantly larger than those in stop trials during about 200–300 ms (*p*<.05 with FDR control; q*<.05), which corresponds to about 150–250 ms after re-go-signal onset, while there was no large cluster with significant PLI differences between stop and re-go(150) trials (Figure 5C).

**Figure 6:**
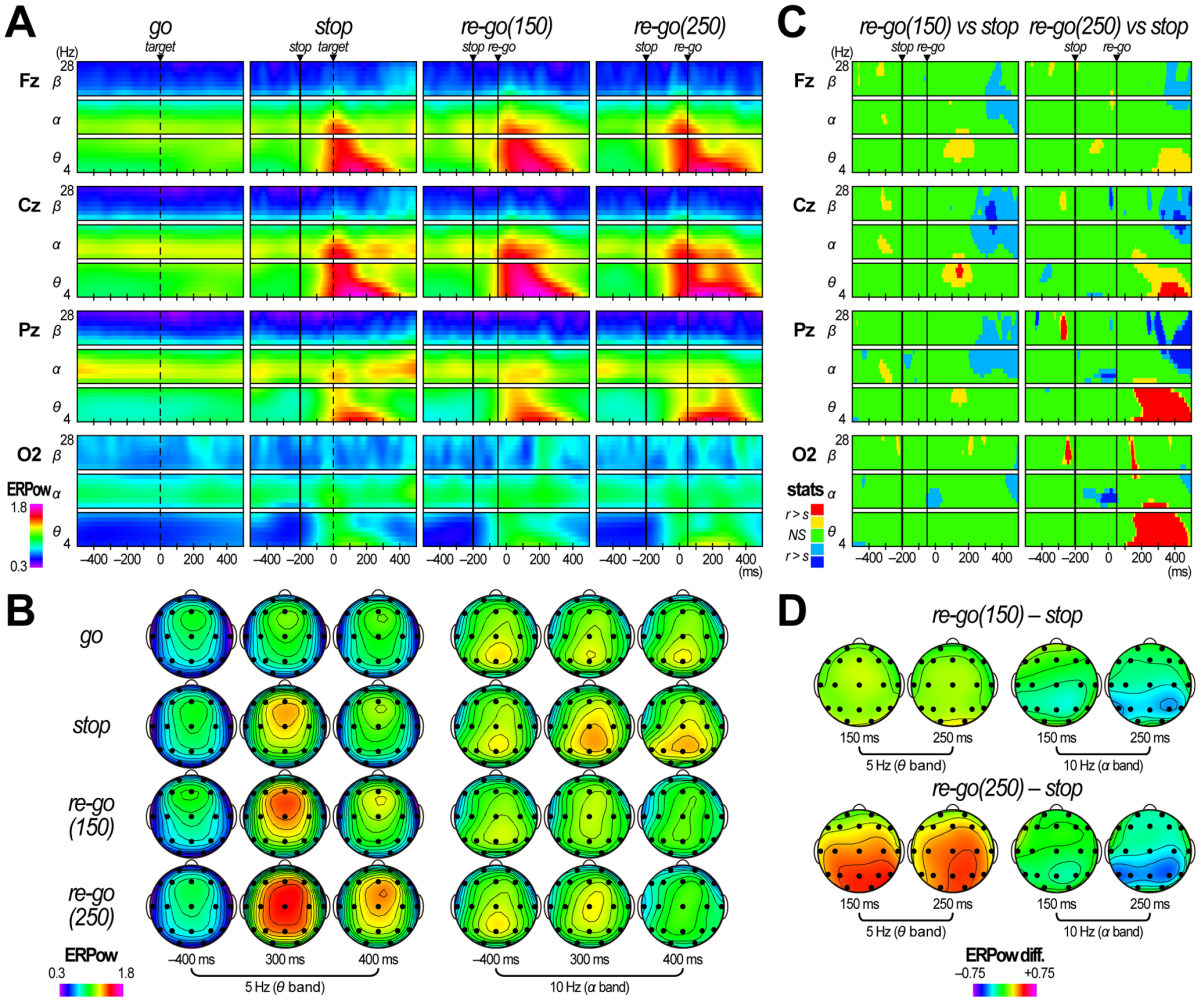
Results of event-related power analysis in Experiment 3. (**A**) Time/frequency (T/F) images of grand mean event-related power (ERPow) obtained during go trials, stop trials, re-go trials with an SRSI of 150 ms (re-go(150)), and re-go trials with an SRSI of 250 ms (re-go(250)). (**B**) Scalp topographies of grand mean ERPow in go, stop, re-go(150), and re-go(250) trials. They are displayed at time/frequency points described in the figure. (**C**) T/F images indicating the results of the ERPow statistical tests (re-go(150) vs. stop and re-go(250) vs. stop). (**D**) Scalp topographies of grand mean ERPow differences (re-go(150) – stop and re-go(250) – stop). They are displayed at time/frequency points described in the figure.

When we compared grand mean ERPow T/F images in go, stop, re-go(150), and re-go(250) trials, large alpha-band ERPow levels were observed throughout the experimental period, mainly over the centroparietal sites (Figure 6A, B), indicating the existence of ongoing alpha-band oscillations. However, the alpha-to-beta-band ERPow increased in stop trials from about 200 ms, whereas it decreased from about 200 ms in re-go(150) trials and from about 300 ms in re-go(250) trials (Figure 6A, B). These alpha-to-beta-band ERPow modulations are similar to those reported in go/no-go studies [22], [28], [29]. Consequently, the alpha-to-beta-band ERPow values at Cz in re-go(150) and re-go(250) trials were significantly smaller than those in stop trials from about 200 and about 300 ms, respectively (*p*<.05 with FDR control; q*<.05) (Figure 6C). These results indicate that re-go–specific decreases occurred in alpha-to-beta-band EEG power over the centroparietal sites after re-go responses were executed. In contrast, the transient theta-band ERPow increases were only prolonged in re-go(250) trials. Consequently, during 200–500 ms, the theta-band ERPow values at Cz, Pz, and O2 in re-go(250) trials were significantly larger than those in stop trials (*p*<.05 with FDR control; q*<.05) (Figure 6C). Such prolongation of the theta-band ERPow increases appeared prominently over the parieto-occipital sites (Figure 6D).

**Figure 7:**
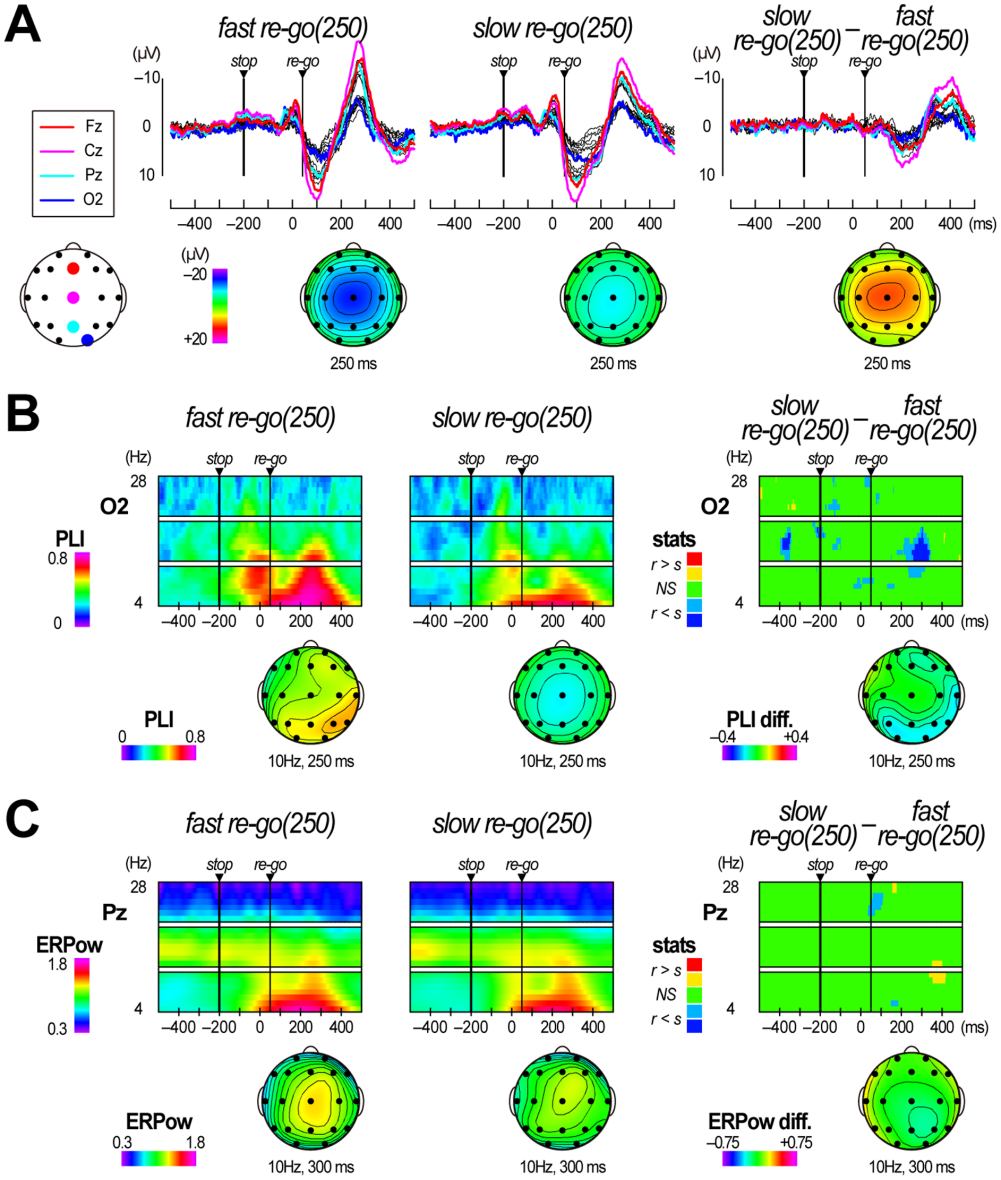
Comparisons of electroencephalographic data between fast and slow re-go(250) trials in Experiment 3. (**A**) Grand mean event-related potential (ERP) waveforms obtained during fast and slow re-go trials with an SRSI of 250 ms (fast re-go(250) and slow re-go(250)) and grand mean ERP difference waveforms (slow re-go(250) – fast re-go(250)). Scalp topographies at 250 ms relative to the target are displayed below each panel. (**B**) Time/frequency (T/F) images of grand mean phase locking index (PLI) obtained during fast re-go(250) and slow re-go(250) trials, and T/F images indicating the results of the PLI statistical test (fast re-go(250) vs. slow re-go(250)). Scalp topographies at 10-Hz/250-ms are displayed below each T/F image. (**C**) T/F images of grand mean event-related power (ERPow) obtained during fast re-go(250) and slow re-go(250) trials, and T/F images indicating the results of the ERPow statistical test (fast re-go(250) vs. slow re-go(250)). Scalp topographies at 10-Hz/300-ms point are displayed below each T/F image.

The mean RTs of dual-partitioning re-go(250) trials were 259.4±24.2 ms (fast re-go(250) trials) and 341.6±25.8 ms (slow re-go(250) trials), indicating that participants performed a stop-and-restart smoothly in the fast re-go(250) trials, but with difficulty in the slow re-go(250) trials. When we compared grand mean ERP waveforms in fast re-go(250) and slow re-go(250) trials (Figure 7A), negative-positive ERP deflections after stop-signal onset were almost the same. However, second negative peaks after re-go-signal onset were earlier and steeper in fast re-go(250) trials than in slow re-go(250) trials. These results indicate that the re-go–specific ERP components differed between fast re-go(250) and slow re-go(250) trials. When we compared grand mean PLI T/F images between fast re-go(250) and slow re-go(250) trials (Figure 7B), second theta-to-alpha-band PLI increases over the parieto-occipital sites were more noticeable in fast re-go(250) than slow re-go(250) trials. Consequently, during about 200–300 ms, the alpha-band PLI values at O2 in fast re-go(250) trials were significantly larger than those in slow re-go(250) trials (*p*<.05 with FDR control; q*<.05). These results indicate that more powerful re-go–specific EEG phase locking appeared in fast re-go(250) trials. In contrast, when we compared grand mean ERPow T/F images between fast re-go(150) and slow re- go(250) trials (Figure 7C), we could not detect any clear differences in ERPow levels.

## Discussion

To assess the difficulty of stop-and-restart movement, we first examined performance in re-go trials with four different SRSIs in Experiment 1. With respect to the RTs in SRT task trials, mean re-go RTs with the shortest (100 ms) and the two longest (300 and 400 ms) SRSIs were delayed, whereas those with a moderate SRSI (200 ms) were comparable (Figure 2C, left and top right). This V-shaped change in mean re-go RTs as a function of SRSI is consistent with results obtained in a previous study [5], and extend the result to a wider range of SRSIs (100–400 ms). In addition, a linear change in SD of re-go RTs as a function of SRSI was observed (Figure 2C, left and bottom right). That is, compared with the distribution of RTs in SRT task trials, the distribution of the re-go RTs with the shortest SRSI (100 ms) shifted to the slower range, but did not change the shape, and the distribution of the re-go RTs with the two longest SRSIs (300 and 400 ms) became wider in the slower range. Conversely, the distribution of RTs in re-go trials with an SRSI of 200 ms was almost same as that for SRT task trials. These results indicate that consistent stop-and-restart difficulties occur after the shortest SRSI (about 100 ms), and the probability of difficulty increases after longer SRSIs (>200 ms), whereas stop-and-restart movement can be performed smoothly just after an appropriate SRSI (about 200 ms). Moreover, these results suggest that two mechanisms underlie stop-and-restart difficulties: one acts after the short SRSI and slows RTs consistently, and the other acts mainly after the longer SRSIs and slows RTs stochastically. Therefore, we examined the mechanisms underlying stop-and-restart difficulties in two electrophysiological experiments.

In Experiment 2, we observed biphasic MEP modulation (initial decrease and later increase) in re-go trials; this indicates stop-related suppression and pre-(re-go) movement enhancement of CM excitability (Figure 3A and B, middle and bottom). The temporary stop-related suppression in CM excitability in re-go trials can be taken as physiological evidence for a hidden, transient stop process. We also found that the pre-movement enhancement of CM excitability was delayed longer, after re-go-signal onset, in re-go trials with an SRSI of 100 ms than in re-go trials with an SRSI of 200 ms (Figure 3C). TMS time of 100 ms after re-go-signal onset in re-go trials with an SRSI of 100 ms corresponds exactly to the target time of the primary go trials (Figure 3A and B). At that time point, CM excitability was strongly suppressed in both stop and re-go trials. Many previous studies have reported pre-movement enhancement of CM excitability within 100 ms before response onset [11], [12], [30]-[32]. In re-go trials with an SRSI of 100 ms, starting a motor preparation 100 ms after re-go-signal onset might be difficult because stop-related motor suppression is active during that time. In contrast, in re-go trials with an SRSI of 200 ms, starting a motor preparation 100 ms after re-go-signal onset might be relatively easy because the stop-related motor suppression might have nearly ceased by then. Taken together, the results of Experiment 2 suggest that stop-and-restart difficulties with a short SRSI (100 ms) might be associated with stop-related suppression of CM excitability. In contrast, stop-and-restart difficulties with longer SRSIs cannot be explained by the suppression of CM excitability because the MEP amplitudes in stop trials of the go/stop/re-go tasks did not decrease at the longer TMS times (100–200 ms after re-go-signal onset, Figure 3A and B, bottom).

In Experiment 3, we observed negative-positive ERP deflections in ERP waveforms (Figures 4 and 7A), commonly occurring in stop and all types of re-go trials, which can serve as additional physiological evidence that an unobservable stop process is active in all types of re-go trials. In contrast, additional ERP components were observed only in re-go trials, suggesting the existence of re-go–specific EEG dynamics. Moreover, in the single-trial–based EEG power (ERPow) and phase (PLI) T/F maps, we observed re-go–specific modulations that were separated into three group: 1) theta-band power and phase dynamics, 2) alpha-band phase dynamics, and 3) alpha-to-beta band power dynamics. We discuss them in turn, below.

First, the theta-band ERPow and PLI increases were prolonged to about 400 ms only in the re-go(250) trials (Figure 5 and 6). Previous studies have reported that parallel ERPow and PLI increases in theta band likely reflect the existence of polarity- and latency-fixed deflections, the cycle-length of which correspond to the theta band, and suggested that these are the significant underlying bases of the typical ERP waveforms [22], [33]. In fact, we observed distinct prolongation of large ERP waveforms in the re-go(250) trials (Figure 4), and significant ERPow and PLI differences were only observed between stop and re-go(250) trials (Figure 5 and 6). This prolongation of theta-band EEG dynamics might be associated with the time required for task operation [34].

Second, the second transient alpha-band PLI increases only appeared over the parieto-occipital sites in re-go(250) trials (Figure 5). Previous studies have reported alpha-band PLI increases related to an early ERP component over the parieto-occipital sites, and have suggested that they might reflect visual perception [16], [18], . That is, in re-go(250) trials, participants might perceive the re-go-signal separately from the preceding stop-related processes, and execute stop-and-re-go response as two separate tasks. In contrast, in re-go(150) trials, they might perceive the re-go-signal within the stop-related process, and execute the stop-and-re-go responses as a single task. Therefore, RTs in re-go(250) trials were longer than those in re-go(150) trials because it takes time for the second visual perception to occur. Moreover, alpha-band PLI increases over the parieto-occipital sites were larger in fast re-go(250) trials than in slow re-go(250) trials (Figure 7B). This indicates that participants could perceive the re-go-signal well in a majority of fast re-go(250) trials, while they could perceive it well only in a minority of slow re-go(250) trials. These results suggest that stop-and-restart difficulties with long SRSIs might be related to perceiving the re-go-signal as a second task.

Third, the alpha-to-beta-band ERPow changes were in the opposite directions for stop and re-go trials (increase and decrease, respectively). Moreover, the alpha-to-beta-band ERPow decreases appeared slightly earlier in re-go(150) trials than re-go(250) trials (Figure 6). Previous studies have reported alpha-to-beta-band EEG power decreases (which are analogous to event-related desynchronization) after the motor response over the centro-parietal sites, and have suggested that this decrease might reflect motor execution and its derivative process [22], [28], [29], . Other studies reported that alpha-band EEG power increases after motor suppression, and suggested that this might relate to motor inhibition and its derivative process [22], [28], [29]. Our results are in agreement with these previous studies. In contrast, there was no significant difference in alpha-to-beta-band ERPow between fast and slow re-go(250) trials (Figure 7C). This inconsistency might simply be due to the differences in comparisons (i.e., stop vs. re-go or re-go vs. re-go). In fact, early ERPow decreases in fast re-go(250) trials could be observed in scalp topography for ERPow differences in the alpha band (Fig. 7C, bottom right).

Considering all of the EEG data together, we suggest that stop-and-restart difficulties after long SRSIs may be associated with failure of visual perception, which is reflected in alpha-band phase dynamics. As a result, the re-go response is delayed and the time to complete the task is prolonged, which are reflected in theta-band power and phase dynamics and alpha-to-beta-band power dynamics, respectively. Although the precise cause of the failure of visual perception is still unknown, it is thought to be a problem in the attention system, not in the perception system alone. An index of explicit attentional breaks in a task is eye-blink [44]. During EEG data preprocessing, we confirmed that only a small number of trials were contaminated by large eye-blink potentials. This indicates that participants did not blink during task performance, at least, within the EEG-analysed epoch, suggesting that stop-and-restart difficulties with long SRSIs were not caused by eye blinks. However, we cannot rule out the possibility that the attention system is implicitly associated with the failure of visual perception in the delayed re-go response trials, similar to the attentional blink [45]. Moreover, it remains an open question whether the perception (and attention) problem is caused by preceding strong motor suppression.

Some methodological limitations of this study need to be mentioned. First, the number of TMS trials per condition in Experiment 2 was very low (3 to 5 trials were used for the MEP analysis). MEP amplitude has large variability [46], and mean values from such few trials be easily affected by outliers. The small number of trials was due to the time limitation of the TMS experiment (taking about 1.5 hour to complete) to maintain participants' arousal and attention to the task. In this study, the percentage of trials with TMS in one block of the go/stop/re-go task was small (50/120) because of the complex task structure. In addition, the inter-trial interval needed to accommodate the TMS system recharging time was relatively long (6 s). Therefore, we could not execute more than five trials per time point in this study. Although significant modulations of mean MEP amplitudes were observed even in such disadvantageous conditions, we need to interpret them carefully. Second, this was a combination study of three experiments with slightly different task settings. Therefore, such slight differences in task settings might affect the results. For example, mean go RTs in both go/stop tasks in Experiment 1 and 2 tended to be larger than those reported in previous studies [25], [26]. This might be mainly due to large proportion of the stop signal presentation (50%), which was chosen for this experiment in order to execute as many re-go trials as possible. Next, in Experiment 1, mean go RTs in the go/stop task were significantly closer to the target than those in the go/stop/re-go tasks, as described above. This might be mainly due to large potential for response execution in the go/stop/re-go tasks. Thus, we need to keep in mind that the results of Experiment 2 (MEP) and 3 (EEG) may be influenced by these slight differences in task settings.

In summary, we investigated stop-and-restart movement performance by using a timing-coincident go/stop/re-go task. We found consistent stop-and-restart difficulties after the shortest SRSI (100 ms), and an increased probability of stop-and-restart difficulties after longer SRSIs (>200 ms), suggesting that two different mechanisms underlie the difficulties associated with stop-and-restart movements. In an attempt to identify these mechanisms, we examined MEPs induced by TMS, as well as EEG dynamics, in re-go trials. Consequently, stop-and-restart difficulties are associated with stop-related suppression in CM excitability in re-go trials with short SRSIs, whereas they are associated with decreased re-go-signal perception (reflected in the weakened EEG phase locking to visual stimuli) in re-go trials with long SRSIs. In contrast, smooth stop-and-restart movement can be achieved in re-go trials with proper SRSI (150-200 ms), because there may be a release from stop-related suppression in CM excitability, and simultaneous counter-activation as a single task without second re-go-signal perception. Moreover, these results suggest that human skilled motor behavior is subject to various constraints in motor and perceptual (and attentional) systems.
